# Surprisal Analysis of Glioblastoma Multiform (GBM) MicroRNA Dynamics Unveils Tumor Specific Phenotype

**DOI:** 10.1371/journal.pone.0108171

**Published:** 2014-09-29

**Authors:** Sohila Zadran, Francoise Remacle, Raphael Levine

**Affiliations:** 1 Institute of Molecular Medicine, David Geffen School of Medicine, University of California Los Angeles, Los Angeles, California, United States of America; 2 Department of Chemistry, B6c, University of Liege, Liege, Belgium; 3 Crump Institute for Molecular Imaging and Department of Molecular and Medical Pharmacology, David Geffen School of Medicine, University of California Los Angeles, Los Angeles, California, United States of America; 4 Institute of Chemistry, Hebrew University of Jerusalem, Jerusalem, Israel; University of Torino, Italy

## Abstract

Gliomablastoma multiform (GBM) is the most fatal form of all brain cancers in humans. Currently there are limited diagnostic tools for GBM detection. Here, we applied surprisal analysis, a theory grounded in thermodynamics, to unveil how biomolecule energetics, specifically a redistribution of free energy amongst microRNAs (miRNAs), results in a system deviating from a non-cancer state to the GBM cancer –specific phenotypic state. Utilizing global miRNA microarray expression data of normal and GBM patients tumors, surprisal analysis characterizes a miRNA system response capable of distinguishing GBM samples from normal tissue biopsy samples. We indicate that the miRNAs contributing to this system behavior is a disease phenotypic state specific to GBM and is therefore a unique GBM-specific thermodynamic signature. MiRNAs implicated in the regulation of stochastic signaling processes crucial in the hallmarks of human cancer, dominate this GBM-cancer phenotypic state. With this theory, we were able to distinguish with high fidelity GBM patients solely by monitoring the dynamics of miRNAs present in patients' biopsy samples. We anticipate that the GBM-specific thermodynamic signature will provide a critical translational tool in better characterizing cancer types and in the development of future therapeutics for GBM.

## Introduction

Glioblastoma multiforme (GBM) is an aggressive primary brain tumor that exhibits extremely poor response to current therapies. Despite maximal therapy with surgical resection, radiation and temozolomide, survival statistics remain dismal [1], [2]. According to the National Brain Tumor Society, GBM accounts for approximately 23 percent of all primary brain tumors diagnosed in the U.S. The prognosis for survival beyond the five-year mark is poor, and the average survivability for people diagnosed with GBM is one year [3]. Early diagnosis and treatment however often extend the quality and length of life for individuals suffering with GBM. Early detection of high-grade malignancy also significantly increases the treatment options available and patient survival rate [4]. Several studies have attempted to identify potential biomarkers for GBM. In a study of 125 GBM patients, MGMT promoter methylation was strongly correlated to GBM survival [5]. In addition to EGFR amplification, maintenance of PTEN, wild-type p53 and p16 has also been associated with improved survival in GBM following chemotherapy [6]. Loss of heterozygosity (LOH) on chromosome 10q23 in primary GBMs and TP53 mutations in secondary GBMs has also been well documented in patient studies [7]. Studies using microRNA (miRNA) arrays and glioma tissues found that miR-27a was up-regulated in the glioma cell lines and patients samples by quantitative real-time polymerase chain reaction (qRT-PCR) and suggest that miR-27a may be implicated in the progression of glioma through the modulation of neurotrophin signaling pathway, the MAPK signaling pathway, the transforming growth factor-β (TGF-β) signaling pathway, cytokine-cytokine receptor interactions, the p53 signaling pathway, the apoptotic signaling pathway, as well as others [8]. The stable expression of a targeting construct against miR-27, an anti-miR-27 construct, significantly reduced the proliferation and the accumulation of U87 GBM cells and impaired the invasiveness of U87 GBM cells *in vitro* [9]. However, the prognostic value of these biomarkers has yet to be ascertained and further progress needs to be made not only to elucidate the mechanisms underlying the role of these biomarkers in GBM cancer progression but also to identify others.

MiRNAs are single-stranded short coding RNA molecules of approximately 22 nucleotides in length. MiRNAs guide the RNA-induced silencing complex (RISC) to post-transcriptionally repress the expression of protein-coding genes by binding to targeted messenger RNAs (mRNAs). MiRNAs constitute only 1–3% of the human genome, yet are estimated to control approximately one third of all gene expression. A single miRNA has been observed to control over 100 target mRNAs [10]. Conversely, a single mRNA can be modulated by multiple miRNAs. Over 1,000 miRNAs have been identified in humans according to a registry (miRBase) cataloguing all reported discoveries. MiRNAs play crucial regulatory roles in several cellular processes, including growth, proliferation, metabolism, development, and apoptosis [11], [12]. Given miRNAs widespread regulatory function within the cell, the aberrant expression of miRNAs has naturally been implicated in several human diseases, such as diabetes, arthritis, kidney disease, neurodegenerative disorders and cancer [13]. Studies of genome-wide miRNA expression have indicated that a majority of human miRNA genes are located at fragile genomic sites associated with cancer, and that miRNAs can function both as an oncogene and as a tumor suppressor. However, understanding how miRNA dynamics differ between phenotypic states on a systems level has remained an enigma, particularly in understanding how global modulations of miRNAs can program a non-cancer state to exhibit cancer-phenotypic characteristics. Currently there exist several computational miRNA target prediction tools, which are heavily dependent on complementarily to miRNA seeds and evolutionary conservation. Although these features allow for successful target identification, not all miRNA target sites are conserved and adhere to canonical seed complementarity.

We applied an unbiased thermodynamic maximal-entropy based approach, known as surprisal analysis, to examine global miRNA expression dynamics of 534 miRNAs in over 490 GBM patients. This theoretical analysis unveils a GBM-specific miRNA thermodynamic signature capable of distinguishing healthy and GBM patients with high fidelity. Surprisal analysis also identified a miRNA system response unique to GBM patients, where miRNAs implicated in the regulation of stochastic signaling processes crucial in the hallmarks of human cancer, including cell proliferation and cancer metabolism, contributed the greatest free-energy to sustaining the GBM-cancer phenotypic state. We anticipate that the GBM-specific miRNA signature introduced here will have substantial translational potential and utility as a high-throughput drug discovery platform, particularly in better understanding how current therapeutics can affect biomolecule dynamics in human cancers.

## Results

### Surprisal analysis identifies a thermodynamic miRNA signature unique to GBM

Significant effort has been made in identifying disease signatures, individual genes or compilations of modulated genes that can be used to characterize the phenotypic states of disease [14]–[16]. Surprisal analysis was applied to the miRNA microarray expression profiles collected from 490 GBM patients' tumor tissue samples and compared to the expression profiles from 10 non-GBM healthy controls. Surprisal analysis begins with the identification of a reference defined as the balance state, in which all cellular processes are assumed at equilibrium and therefore for which there is no net change in the system, as previously described by [17]. It is an idealized reference state common to both healthy and diseased patients. Then, constraints that deviate the system away from this balance state are determined and characterized. These constraints unveil observable phenotypic cell states unique to the system. We not only identify these constraints but also characterize the signatures, specifically biomolecule dynamics that are responsible for the deviation of the system from the balance state into phenotypic states. In the present analysis the by far dominant signature is the GBM-specific cancer state. The theoretical analysis quantifies the importance of each miRNA in each of the system's signatures.

**Figure 1:**
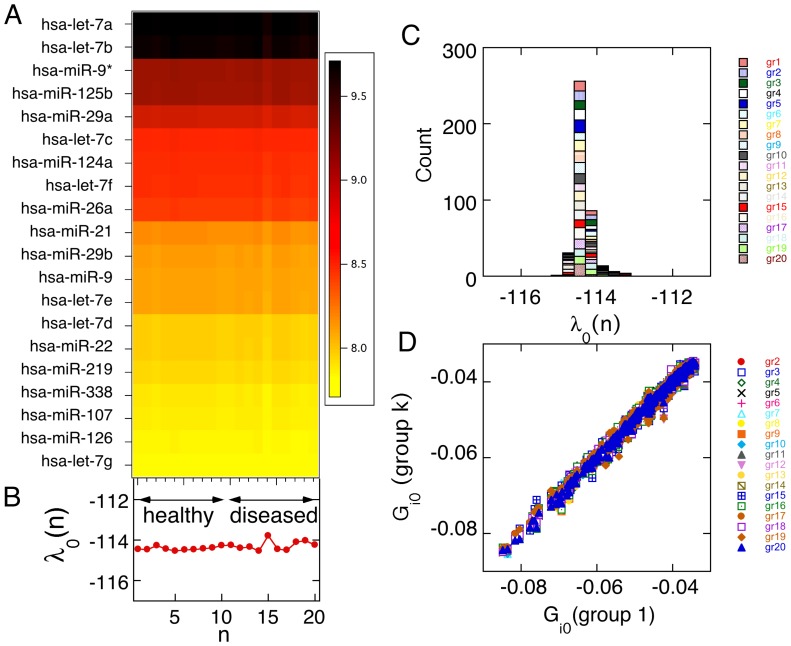
The balance state is common to GBM diseased and healthy patients. The heat map A is a representation that highlights the invariance across patients where each column is an individual patient. Each row is a different miRNA. The miRNAs with the greatest contribution to the balance state are listed in order of descending contribution (and decreasing energetic stability, on a ln scale, see inset on the right) where dark is more stable and yellow is less stable. In the balance state, all patients are exhibiting similar expression pattern. B. The plot shows the patient potential in the balance state ( =  Lagrange multiplier for the balanced state), as described in the methods, vs. the patient index, *n*, coinciding with the heat map in A. No significant variation of is observed between healthy and GBM diseased patients, as expected for a balance state that is common to both GBM and normal patients. C. An alternative graphical representation of the stability of the balanced state. Shown is a histogram of the patient potentials in the balanced state, computed for the 10 healthy patients and 20 groups of 10 diseased patients each, showing altogether 200 diseased patients. The histograms is a rather narrow peak, indicating a common value to both healthy and diseased patients. The range of the ordinate in B is the same as the range of the abscissa used in the histogram. D. Signatures of the balance state of 19 different patient groups, 2 to 20, are shown in the legend as a scatter plot vs. the signature of patients group 1. Each group has 10 healthy patients and different sets of 10 diseased ones. Despite patient variability the signatures of the different groups are very consistent across the entire range of (only negative) possible values of G_i_.

The list of miRNAs with largest negative values of G_i_ is shown in Figure 1. These are the most stable ( =  lowest free energy) miRNAs that sustain the balance state. We find that the largest contribution of free energy of individual miRNAs to the balance state is from miRNAs networks involved in maintaining cellular homeostasis and evolutionary conserved miRNAs. Furthermore, both GBM systems and normal systems show a very similar distribution of standard free energy amongst miRNAs in the balance state, suggesting that GBM cancer and normal systems may share a common thermodynamic lineage (Figures 1A and 1C). Heat maps of the balance state demonstrate, as expected, that the expression flux of miRNAs that comprise the balance state is consistent across all patients. Therefore the balance state is robust against patient variability (Figure 1B and Figure 1 D). We anticipate that slight variability (≤10%) between GBM patients may arise from varying amounts of necrotic or dead cells in the heterogeneous tumor samples prior to microarray processing. We previously investigated the role of experimental noise by examining the balance state at different points in time during carcinogenesis of a homogenous cell population *in vitro* [18].

In the balance state, we observe the let-7 family of miRNAs; let-7 and its family members are highly conserved across species in sequence and function, and misregulation of let-7 leads to a less differentiated cellular state [19]. Additionally, we see miR-22 in the balance state, also highly conserved across many vertebrate species. MiR-22 directly targets histone deacetylases involved in DNA replication and transcription [20]. Interestingly, we also see miR-9*, a miR highly expressed in the normal brain and critically involved in the neuronal differentiation and basic neuronal function [21] present in the balance state, suggesting that the balance state not only consists of miRNAs that regulate the basic cellular functions but also are tissue specific (Table S1).

Many of miRNA functions remain unknown and the mechanisms driving cooperative regulation between miRNAs and their cellular targets are also still not fully understood. MiRNAs tend to target highly connected genes in cellular networks. Additionally, the miRNAs that constitute the balance state are directly related to the regulation and expression of components of the cellular homeostasis system, such as chaperones and the cellular machinery involved in the proteasome, cell cycle, autophagy and cell transport. The health of a cell is inextricably correlated to cellular quality control, where a highly complex network of molecular interactions balances protein synthesis, protein function, cellular metabolism and protein clearance. Deviations from cellular homeostasis or aberrations in the machinery that regulates processes associated with cellular homeostasis results in global, system-wide decline in cellular function, with deleterious consequences on tissue and ultimately patient viability [22]. Therefore, we sought out to determine the miRNAs that contributed to a system deviating from the balance state to a cancer specific phenotypic state.

**Figure 2:**
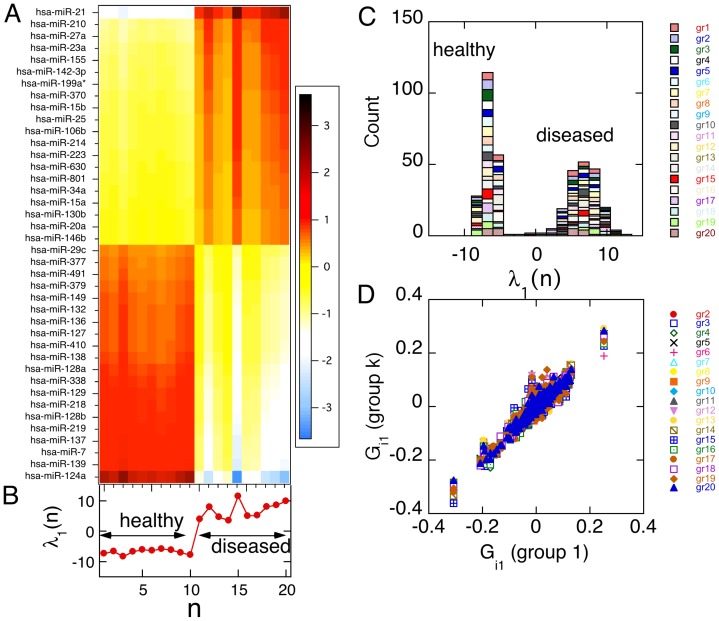
The GBM cancer-specific miRNA thermodynamic signature distinguishes GBM and healthy patients. A. The heat map shows the miRNAs with the greatest contribution to the GBM cancer phenotypic state, up regulated and down regulated with respect to the balanced state (on a ln scale, see colour code in the inset). Similar thermodynamic behavior is observed across patients, however patient specific variability is observed. B. The plots shows the patient potential in the disease signature ( =  first Lagrange multiplier), on the same scale of patient index, *n*, coinciding with the heat map in A. Distinct difference in sign of the lagrange multiplier is observed between healthy and GBM diseased patients, delineating the two phenotypic states. C. A histogram of the patient potentials in the disease signature computed for the 10 healthy patients and 20 groups of 10 diseased patients each, showing altogether 200 diseased patients. D Different groups of diseased patients have consistent signatures in both balance state and disease. Disease signatures of 19 different patient groups, 2 to 20, are shown as a scatter plot vs. the signature of patients group 1. Each group has 10 healthy patients and a different set of 10 diseased ones. The groups are identified in the legend. Despite patient variability the signatures of the different groups are very consistent across the entire range of possible values of G_i_.

After determining the miRNAs that contribute to sustaining the balance state, we next identify the constraints placed on the system when deviating away from the balance state. Surprisal analysis identifies the largest deviation away from the balance state and in our analysis, the largest deviation from the balance state is revealed to be a unique signature of the disease state. This constraint is capable of distinguishing healthy and GBM patient samples and is characterized as specific to the GBM cancer phenotypic state. We also determine the miRNAs with the greatest contribution to this GBM cancer phenotypic state and utilize this miRNA signature to robustly distinguish between healthy and GBM cancer patients (Figure 2A). In addition, the analysis characterizes degrees of patient variability, or the thermodynamic potentials of the disease for each patient (Figure 2B and Figure 2C). These patient-specific potentials in the cancer phenotypic state may provide an avenue for development of personalized diagnostics.

In the GBM-specific signature, we identified miRNAs that regulate critical signaling machinery involved in cell proliferation, cancer invasiveness and directly affect GBM cancer aggressiveness (Table S2 and Table S3). For instance, we observe miR-21 to have the greatest positive free –energy contribution to deviating a system from a non-GBM state to the GBM cancer state (Table S3 and Fig 2A). Previous studies have revealed elevated miR-21 levels in human glioblastoma tumor tissues, early-passage glioblastoma cultures, and in six established glioblastoma cell lines (A172, U87, U373, LN229, LN428, and LN308) compared with nonneoplastic fetal and adult brain tissues and compared with cultured nonneoplastic glial cells. Additionally, knockdown of miR-21 in cultured glioblastoma cells triggers activation of caspases and leads to increased apoptotic cell death [23]. Conversely, in the GBM-specific signature, we identified miR-124 with the largest negative free-energy contribution to deviating a system from the GBM cancer state to the non-cancer state (Table S2 and Fig 2A). Studies have implicated miR-124 as a brain-enriched miRNA that plays a crucial role in neural development and is down regulated in glioma and medulloblastoma, suggesting its possible involvement in brain tumor progression. Additionally, miR-124 is down regulated in a panel of different grades of glioma tissues and in human glioma cell lines [24].

Several studies have suggested that miRNA expression behavior can provide a more accurate method of classifying cancer subtypes than transcriptome profiling of an entire set of known protein-coding genes. Differential miRNA expression behavior has been able to successfully classify poorly differentiated cancers, while mRNA gene expression behavior failed to classify them [25]–[28]. Further characterization and understanding of these miRNA expression behaviors and extension of surprisal analysis to miRNA datasets will lead to the development of tissue- and biofluid-specific diagnostic markers, as well as a new type of oligonucleotide-based therapeutics. We further experimentally validated the GBM-specific signature by application of surprisal analysis to miRNA microarray expression profiles (Agilent) from a cohort of 24 GMB patients. These GBM patients were examined and confirmed by the UCLA Neuro-Oncology and Pathology Departments for high-grade GBM (Table S4). Additional signatures may be identified by surprisal analysis (*see methods*) of expression level data. The second signature is about half as large as the disease signature. Even so, sorting between healthy and diseased patients is clear and robust as shown in Figure 2.

## Discussion

Cancer cells frequently exist in varying phenotypic states and phenotypic equilibrium was observed in cell state-proportions *in vivo* and *in vitro*. Additionally, current anti-cancer therapies preferentially target specific cancer cell states and initiate changes in phenotypic properties of tumors [29]. Therefore further understanding of how cell states are determined, specifically how a normal cell adopts a new phenotypic state and how this state can differ for different patients is of considerable interest and will facilitate the development of targeted and more effective therapeutics. Surprisal analysis is an information-theoretic approach grounded in thermodynamics and is capable of characterizing biomolecule dynamics, such as miRNAs, based on the expression flux of the biomolecule. We anticipate that understanding these biomolecule dynamics will enable us to better monitor bioenergetics of the system. Surprisal analysis has already been applied to a spectrum of disciplines including engineering, physics, chemistry and biomedical engineering and is here used to thermodynamically characterize biological systems based on biomolecule cellular dynamics.

We apply surprisal analysis to elucidate a GBM-cancer specific miRNA signature. This signature was able to distinguish between healthy and GBM patients. The miRNAs in this signature have been implicated in cancer progression. Aberrant expression of microRNA (miRNA) is commonly associated with cancer and loss of miR-124 has previously been implicated to function as a tumour suppressor. The expression levels of mature miR-124 in a retrospective series of 119 cases of histologically confirmed GBM and found its expression was markedly lower in over 80% of the GBM clinical specimens compared to normal brain tissue [30]. MiR-21 is up-regulated in different human cancers including glioblastoma, prostate and breast cancer. In addition, mir-21 contains oncogenic properties for it has the ability to negatively modulate the expression of tumor-suppressor genes [31]. Suppression of miR-21 *in vitro* suppressed both cell growth *in vitro* and tumor growth in the xenograft mouse models [32]. MiR-21 was one of the most frequently up regulated miRNA in both neuronal and GBM cell lines [33]. Antisense-miR-21-treated cells showed a decreased expression of EGFR, activated Akt, cyclin D, and Bcl-2 [34]. Consistent with our analysis, previous studies have also revealed the down-regulation of miR-451 in A172, LN229 and U251 human GBM cells. Increased expression of miR-451 by administration of miR-451 mimics oligonucleotides reversed the biology of each of the three cell lines, inhibiting cell growth, inducing G0/G1 phase arrest and increasing cell apoptosis. Further, treatment with miR-451 mimics oligonucleotides diminished the invasive capacity of these cells, as the number of cells invading through matrigel was significantly decreased [35]. MiR-7 is also found to be a tumor suppressor in GBM, targeting critical cancer pathways. MiR-7 potently suppressed epidermal growth factor receptor (EGFR) expression, and furthermore it independently inhibited the Akt pathway via targeting upstream regulators [36]. Patients' overall survival (OS) and progression-free survival (PFS) associated with interested miRNAs and miRNA-interactions as performed by Kaplan-Meier survival analysis of over 400 GBM patients indicated that that low levels of miR-155 and miR-210 were significantly associated with long OS of GBM patients, and also showed that high miR-326/miR-130a and low miR-155/miR-210 were related with extended PFS [37]. Consistent with many miRNAs present in our GBM-specific signature, we find that targeting these miRNAs *in vitro* and *in vivo* may provide an effective therapeutic avenue for GBM. Additionally, we unveil miRNAs critical to the GBM-specific signature that have not yet been examined experimentally, but may expose new miRNA targets in GBM.

We anticipate that application of surprisal analysis and further understanding of patient potentials can also generate predictive formulations to identify biomarkers of cancer that predict individual patients benefit or success rates from a particular targeted therapy. Recent advances in genome technologies and the outpouring of genomic information related to cancer have accelerated the convergence of discovery science and clinical medicine. Our study emphasizes the importance of establishing the biological relevance of a cancer profiling technologies and modeling platforms and exploring the clinical potential and application of their discoveries.

## Methods

### GBM Tissue Preparation

Fresh human glioblastoma specimens (WHO Grade IV) was collected from patients following informed written consent at the David Geffen School of Medicine, University of California, Los Angles. Samples were submitted to the study under a protocol approved by the University of California Office of Human Research Protection and the North General Institutional Review Board (exempt IRB Protocol # 12-001039). Samples were obtained from patients who underwent primary therapeutic subtotal or total tumor resection performed under image guidance. Tissue samples were obtained at primary resection, and none of the patients had undergone prior chemotherapy or radiation therapy. The samples were immediately frozen on dry ice and cut into 12-µm sections. Histological diagnosis of tumor core and invasive edge was made by neuropathological review by standard light-microscopic evaluation of the sections stained with hematoxylin and eosin. The targeted areas were collected by microscopic dissection under an inverted microscope and processed immediately for RNA isolation. Patient miRNA expression analysis will be deposited in GeoAsscession and NCBI.

### RNA extraction, miRNA microarray profiling

Total RNA, including small RNA, was isolated using the mirVana miRNA isolation kit (Ambion, Austin, TX). The RNA quality and quantity were assessed using the NanoDrop 2000 (Thermo Scientific, Waltham, MA). The integrity of RNA was determined using a Bioanalyzer 2100 Nano LabChip kit (Agilent Technologies, Santa Clara, CA). Samples selected for the study contained intact RNA with a RIN≥8.0. One hundred ng total RNA was end-labeled with Cy3-pCp following the manufacturer's recommendations using Agilent's miRNA Complete Labeling and Hyb Kit (Agilent). Labeled miRNA was hybridized to Agilent's Human 8×15K miRNA Microarrays (V2) based on Sanger miRbase (release 10.1). Images were captured using an Agilent DNA Microarray Scanner set at default settings for miRNA microarrays. Scanned TIFF images were processed using Feature Extractor v. 10.5.1.1. Further quality control and normalization was performed using GeneSpring GX 11 (Agilent). Signals <1 were set to 1 due to GeneSpring's analysis in log space, negative values were converted as well. Values were divided by the 75th percentile signal on that array to allow for improved comparison between arrays.

### TCGA miRNA dataset and patient information

Level 3 miRNA expression data were obtained from The Cancer Genome Atlas (TCGA) data portal. MiRNA microarray expression datasets from glioblastoma multiform were acquired in August 2012.

### Surprisal Analysis

Surprisal analysis was utilized to directly compute the probability that a particular patient is diseased as previously described [14], [15]. See also the graphical overview provided in the Schematic S1.

## Supporting Information

Schematic S1 **Surprisal Analysis of GBM Patient Biopsy Samples.** The balanced state is common to all patients, see figure 1 of the main text. The balance state is located at the minimum of the free energy, which is the point of maximal entropy. The disease phenotype causes an increase in the free energy. As seen in figure 2 of the main text, the disease potential has an opposite sign in healthy and diseased patients but about to the same extent in either direction as shown schematically in this figure. The figure shows only one horizontal axis but there can be other axes in orthogonal directions representing additional, secondary, phenotypes, for example representing the distinction between the de novo and the recurrent states.
(DOCX)

Table S1 **miRNAs greatest free energy contribution to the in the balanced state.**
(DOCX)

Table S2 **miRNAs greatest negative free energy contribution to the GBM-specific phenotypic state.**
(DOCX)

Table S3 **miRNAs greatest positive free energy contribution to the GBM-specific phenotypic state.**
(DOCX)

Table S4 **Overlap of signatures between UCLA GBM Cohort and TCGA GBM Cohort.**
(DOCX)

## References

[1] Zhu H, Jaime A, Pranatartiharan R, Abraham B, Steve W, Rolf P, Roderick T Bronson, et al (2009) Oncogenic EGFR signaling cooperates with loss of tumor suppressor gene functions in gliomagenesis Proceedings of the National Academy of Sciences. 106 no.8 2712–2716. 10.1073/pnas.0813314106 PMC2650331 19196966

[2] Joo Kyeung M, Shi Yean K, Xun J, Sang Yong S, Doo-Sik K, Jung-II L, Ji Won J, et al (2008) Clinical and biological implications of CD133-positive and CD133-negative cells in glioblastomas. Laboratory investigation 88 no.8 808–815. 18560366 10.1038/labinvest.2008.57

[3] Verhaak GW, Hoadley K, Purdom E, Victoria W, Qi Y, Wilkerson M, Miller RM, et al (2010) Integrated Genomic Analysis Identifies Clinically Relevant Subtypes of Glioblastoma Characterized by Abnormalities Cancer cell. 17 no.1 98–110. 10.1016/j.ccr.2009.12.020 PMC2818769 20129251

[4] Liang Y, Maximilian D, Watson N, Bollen A, Aldape KD, Nicholas KM, Lamborn KR, et al (2005) Gene expression profiling reveals molecularly and clinically distinct subtypes of glioblastoma multiforme. Proceedings of the National Academy of Sciences of the United States of America 102 no.16 5814–5819. 15827123 10.1073/pnas.0402870102 PMC556127

[5] Phillips H, Samir K, Ruihuan C, Forrest WF, Soriano RH, Wu TD, Misra A, et al (2006) Molecular subclasses of high-grade glioma predict prognosis, delineate a pattern of disease progression, and resemble stages in neurogenesis. Cancer cell 9 no.3 157–173. 16530701 10.1016/j.ccr.2006.02.019

[6] Mischel PS, Shai R, Tao S, Horvath S, Lu KV, Choe G, Seligson D, et al (2003) Identification of molecular subtypes of glioblastoma by gene expression profiling. Oncogene 22 no.15 2361–2373. 12700671 10.1038/sj.onc.1206344

[7] Hegi ME, Diserens AE, Gorlia T, Hamou MF, Tribolet N, Weller N, Kros JM, et al (2005) MGMT gene silencing and benefit from temozolomide in glioblastoma. New England Journal of Medicine 352 no.10 997–1003. 15758010 10.1056/NEJMoa043331

[8] Iorio MV, Croce CM (2009) MicroRNAs in cancer: small molecules with a huge impact. Journal of Clinical Oncology 27 34: 5848–5856. 19884536 10.1200/JCO.2009.24.0317 PMC2793003

[9] Griffiths-Jones S, Harpreet KS, Stijn van D and Enright AJ (2008) miRBase: tools for microRNA genomics. Nucleic acids research 36, no. suppl 1: D154–D158. 10.1093/nar/gkm952 PMC2238936 17991681

[10] Zhang Y, Pengyuan Y, Tao S, Dong L, Xin X, Yaocheng R, Chaoran L, et al (2013) miR-126 and miR-126 repress recruitment of mesenchymal stem cells and inflammatory monocytes to inhibit breast cancer metastasis. Nature cell biology 15 no.3 284–294. 23396050 10.1038/ncb2690 PMC3672398

[11] Li J, Zhang P, Hui N (2012) MiR-218 impairs tumor growth and increases chemo-sensitivity to cisplatin in cervical cancer. International journal of molecular sciences 13 12: 16053–16064. 23443110 10.3390/ijms131216053 PMC3546678

[12] Baffa R, Matteo F, Stefano V, O'Hara B, Chang-Gong L, Palazzo JP, Gardiman M, et al (2009) MicroRNA expression profiling of human metastatic cancers identifies cancer gene targets. The Journal of pathology 219 no.2 214–221. 19593777 10.1002/path.2586

[13] Zhang Y, Pengyuan Y, Tao S, Dong L, Xin X, Yaocheng R, Chaoran L, et al (2013) miR-126 and miR-126* repress recruitment of mesenchymal stem cells and inflammatory monocytes to inhibit breast cancer metastasis. Nature cell biology 15 no.3 284–294. 23396050 10.1038/ncb2690 PMC3672398

[14] Olarerin-G, Anthony O, Lauren A, Yih-Chii H, Michal AE, Hogenesch JB (2013) A functional genomics screen for microRNA regulators of NF-kappaB signaling. BMC biology 11 no.1 19. 23448136 10.1186/1741-7007-11-19 PMC3621838

[15] Graeber TG, Heath JR, Skaggs JB, Phelps ME, Remacle F, Levine RD (2010) Maximal entropy inference of oncogenicity from phosphorylation signaling. Proceedings of the National Academy of Sciences 107 no.13 6112–6117. 10.1073/pnas.1001149107 PMC2851899 20224037

[16] Remacle F, Kravchenko-Balasha N, Levitzki A, Levine RD (2010) Information-theoretic analysis of phenotype changes in early stages of carcinogenesis. Proceedings of the National Academy of Sciences 107 no.22 10324–10329. 10.1073/pnas.1005283107 PMC2890488 20479229

[17] Kravchenko-Balasha N, Remacle F, Gross A, Rotter V, Levitzki A, Levine RD (2011) Convergence of logic of cellular regulation in different premalignant cells by an information theoretic approach. BMC systems biology 5: 42. 21410932 10.1186/1752-0509-5-42 PMC3072338

[18] Kravchenko-Balasha N, Levitzki A, Goldstein A, Rotter V, Gross A, Remacle F, Levine RD (2012) On a fundamental structure of gene networks in living cells. Proceedings of the National Academy of Sciences 109 no.12 4702–4707. 10.1073/pnas.1200790109 PMC3311329 22392990

[19] Zisoulis DG, Zoya SK, Chang RK, Pasquinelli AE (2012) Autoregulation of microRNA biogenesis by let-7 and Argonaute. Nature 486 no.7404 541–544. 22722835 10.1038/nature11134 PMC3387326

[20] Nass D, Shai R, Eti M, Shlomit G, Tabibian-Keissar H, Schlosberg A, Kuker H, et al (2009) MiR-92b and miR-9/9* are specifically expressed in brain primary tumors and can be used to differentiate primary from metastatic brain tumors. brain pathology 19 no.3 375–383. 18624795 10.1111/j.1750-3639.2008.00184.x PMC2728890

[21] Ma L, Young J, Harsha P, Pan E, Mestdagh P, Muth D, Teruya-Feldstein J, et al (2010) miR-9, a MYC/MYCN-activated microRNA, regulates E-cadherin and cancer metastasis. Nature cell biology 12 no.3 247–256. 20173740 10.1038/ncb2024 PMC2845545

[22] Holter NS, Madhusmita M, Amos M, Marek C, Banavar JR, Fedoroff NV (2000) Fundamental patterns underlying gene expression profiles: simplicity from complexity. Proceedings of the National Academy of Sciences 97 no.15 8409–8414. 10.1073/pnas.150242097 PMC26961 10890920

[23] Papagiannakopoulos T, Shapiro A, Kosik KS (2008) MicroRNA-21 targets a network of key tumor-suppressive pathways in glioblastoma cells. Cancer Research 68 19: 8164–8172. 18829576 10.1158/0008-5472.CAN-08-1305

[24] Fowler A, Thomson D, Giles K, Maleki S, Mreich E, Wheeler H, Leedman P, et al (2011) miR-124a is frequently down-regulated in glioblastoma and is involved in migration and invasion. European Journal of Cancer 47 no.6 953–963. 21196113 10.1016/j.ejca.2010.11.026

[25] Haldar S, Basu A (2011) Modulation of MicroRNAs by Chemical Carcinogens and Anticancer Drugs in Human Cancer: Potential Inkling to Therapeutic Advantage. Molecular and cellular pharmacology 3 3: 135. 22288002 PMC3266367

[26] Simon R, Radmacher MD, Dobbin K, McShane LM (2003) Pitfalls in the use of DNA microarray data for diagnostic and prognostic classification. Journal of the National Cancer Institute 95 no.1 14–18. 12509396 10.1093/jnci/95.1.14

[27] Remacle F, Kravchenko-Balasha N, Levitzki A, Levine RD (2010) Information-theoretic analysis of phenotype changes in early stages of carcinogenesis. Proceedings of the National Academy of Sciences 107 no.22 10324–10329. 10.1073/pnas.1005283107 PMC2890488 20479229

[28] Zadran S, Standley S, Wong K, Otiniano E, Amighi A, Baudry M (2012) Fluorescence resonance energy transfer (FRET)-based biosensors: visualizing cellular dynamics and bioenergetics. Applied microbiology and biotechnology 96 no.4 895–902. 23053099 10.1007/s00253-012-4449-6

[29] Gupta PB, Fillmore CM, Guozhi J, Sagi DS, Kai T, Kuperwasser C, Lander ES (2011) Stochastic state transitions give rise to phenotypic equilibrium in populations of cancer cells. Cell 146 no.4 633–644. 21854987 10.1016/j.cell.2011.07.026

[30] Sun J, Xue G, Puro B, Zhao Z (2012) Uncovering microRNA and transcription factor mediated regulatory networks in glioblastoma. PLoS computational biology 8 no.7 e1002488. 22829753 10.1371/journal.pcbi.1002488 PMC3400583

[31] Karsy M, Erol A, Moy F (2012) Current progress on understanding microRNAs in glioblastoma multiforme. Genes & cancer 3 1: 3–15. 22893786 10.1177/1947601912448068 PMC3415667

[32] Hua D, Fan M, Dong D, Li L, Xu H, Zhao H, Foltz G, Lin B, Lan Q, Huang Q (2012) A catalogue of glioblastoma and brain MicroRNAs identified by deep sequencing. mics: a journal of integrative biology 16 no.12 690–699. 10.1089/omi.2012.0069 PMC3521142 23215807

[33] Costa PM, Cardoso AL, Pereira de Almeida LR, Bruce JR, Canoll P, Pedroso de Lima MC (2012) PDGF-B-mediated downregulation of miR-21: new insights into PDGF signaling in glioblastoma. Human molecular genetics 21 no.23 5118–5130. 22922228 10.1093/hmg/dds358 PMC3490519

[34] Costa PM, Cardoso AL, Clévio N, Pereira de Almeida LR, Bruce JN, Canoll P, Pedroso de Lima MC (2012) MicroRNA-21 silencing enhances the cytotoxic effect of the antiangiogenic drug sunitinib in glioblastoma. Human molecular genetics: dds496. 10.1093/hmg/dds496 PMC3561912 23201752

[35] Kim Y, Soyeon R, Lawler S, Friedman A (2011) miR451 and AMPK mutual antagonism in glioma cell migration and proliferation: a mathematical model. PloS one 6 no.12 e28293. 22205943 10.1371/journal.pone.0028293 PMC3243681

[36] Kefas B, Jakub G, Laurey C, Yunqing L, Roger A, Hawkinson M, Jeongwu L, et al (2008) microRNA-7 inhibits the epidermal growth factor receptor and the Akt pathway and is down-regulated in glioblastoma. Cancer research 68 no.10 3566–3572. 18483236 10.1158/0008-5472.CAN-07-6639

[37] Qiu S, Sheng L, Dan H, Yimin F, Yang T, Ying P (2013) Interactions of miR-323/miR-326/miR-329 and miR-130a/miR-155/miR-210 as prognostic indicators for clinical outcome of glioblastoma patients. J Transl Med 11 no.10 10–1186. 23302469 10.1186/1479-5876-11-10 PMC3551827

