# Supplementary material for: Surprisal Analysis of Glioblastoma Multiform (GBM) MicroRNA Dynamics Unveils Tumor Specific Phenotype
Source: PLoS One. 2014 Sep 29;9(9):e108171. doi: 10.1371/journal.pone.0108171 (PMC4180445; doi:10.1371/journal.pone.0108171)
Supplement: Schematic S1 — Surprisal Analysis of GBM Patient Biopsy Samples. The balanced state is common to all patients, see figure 1 of the main text. The balance state is located at the minimum of the free energy, which is the point of maximal entropy. The disease phenotype causes an increase in the free energy. As seen in figure 2 of the main text, the disease potential has an opposite sign in healthy and diseased patients but about to the same extent in either direction as shown schematically in this figure. The figure shows only one horizontal axis but there can be other axes in orthogonal directions representing additional, secondary, phenotypes, for example representing the distinction between the de novo and the recurrent states. (DOCX) [file pone.0108171.s001.docx]

**Supplemental Schematic 1:** Surprisal Analysis of GBM Patient Biopsy Samples


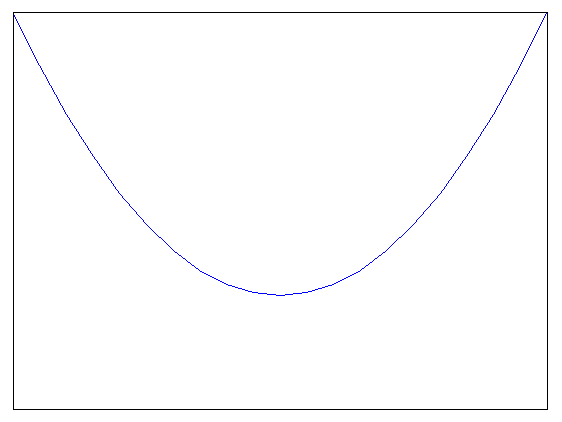


**Constraint Potential**

**Constraint Potential**

**Balance State**

**Healthy State**

**Disease State**

**Free Energy**
