# Supplementary material for: Surprisal Analysis of Glioblastoma Multiform (GBM) MicroRNA Dynamics Unveils Tumor Specific Phenotype
Source: PLoS One. 2014 Sep 29;9(9):e108171. doi: 10.1371/journal.pone.0108171 (PMC4180445; doi:10.1371/journal.pone.0108171)
Supplement: Table S1 — miRNAs greatest free energy contribution to the in the balanced state. (DOCX) [file pone.0108171.s002.docx]

**Table S1:** The miRNAs with the greatest free energy contribution to the *Balance State*. MiRNAs are listed in descending order down the column and continues from left to right.

| **hsa-let-7b** | **hsa-miR-23b** |
| --- | --- |
| **hsa-miR-9*** | **hsa-miR-101** |
| **hsa-miR-125b** | **hsa-miR-195** |
| **hsa-miR-29a** | **hsa-miR-30a-5p** |
| **hsa-let-7c** | **hsa-miR-222** |
| **hsa-miR-124a** | **hsa-miR-15b** |
| **hsa-let-7f** | **hsa-miR-331** |
| **hsa-miR-26a** | **hsa-miR-130a** |
| **hsa-miR-21** | **hsa-miR-100** |
| **hsa-miR-29b** | **hsa-miR-19b** |
| **hsa-miR-9** | **hsa-miR-638** |
| **hsa-let-7e** | **hsa-miR-181b** |
| **hsa-let-7d** | **hsa-miR-30d** |
| **hsa-miR-22** | **hsa-miR-143** |
| **hsa-miR-219** | **hsa-miR-320** |
| **hsa-miR-338** | **hsa-let-7i** |
| **hsa-miR-107** | **hsa-miR-106b** |
| **hsa-miR-126** | **hsa-miR-768-3p** |
| **hsa-let-7g** | **hsa-miR-181a** |
| **hsa-miR-24** | **hsa-miR-342** |
| **hsa-miR-103** | **hsa-miR-149** |
| **hsa-miR-145** | **hsa-miR-99a** |
| **hsa-miR-16** | **hsa-miR-92** |
| **hsa-miR-29c** | **hsa-miR-451** |
| **hsa-miR-125a** | **hsa-miR-23b** |
