# Supplementary material for: Surprisal Analysis of Glioblastoma Multiform (GBM) MicroRNA Dynamics Unveils Tumor Specific Phenotype
Source: PLoS One. 2014 Sep 29;9(9):e108171. doi: 10.1371/journal.pone.0108171 (PMC4180445; doi:10.1371/journal.pone.0108171)
Supplement: Table S2 — miRNAs greatest negative free energy contribution to the GBM-specific phenotypic state. (DOCX) [file pone.0108171.s003.docx]

**Table S2:** The miRNAs with the greatest negative free energy contribution to the *GBM-specific phenotypic state.* MiRNAs are listed in descending order down the column and continues from left to right. (G1 down regulated)

| **hsa-miR-124a** | **hsa-miR-769-5p** |
| --- | --- |
| **hsa-miR-139** | **hsa-miR-432** |
| **hsa-miR-7** | **hsa-miR-33** |
| **hsa-miR-137** | **hsa-miR-487b** |
| **hsa-miR-219** | **hsa-miR-331** |
| **hsa-miR-128b** | **hsa-miR-95** |
| **hsa-miR-218** | **hsa-miR-299-5p** |
| **hsa-miR-129** | **hsa-miR-324-5p** |
| **hsa-miR-338** | **hsa-miR-411** |
| **hsa-miR-128a** | **hsa-miR-381** |
| **hsa-miR-138** | **hsa-miR-154*** |
| **hsa-miR-410** | **hsa-miR-107** |
| **hsa-miR-127** | **hsa-miR-433** |
| **hsa-miR-136** | **hsa-miR-485-3p** |
| **hsa-miR-132** | **hsa-miR-383** |
| **hsa-miR-149** | **hsa-miR-329** |
| **hsa-miR-379** | **hsa-miR-758** |
| **hsa-miR-491** | **hsa-miR-125a** |
| **hsa-miR-377** | **hsa-miR-103** |
| **hsa-miR-29c** | **hsa-miR-222** |
| **hsa-miR-29b** | **hsa-miR-628** |
| **hsa-miR-330** | **hsa-miR-487a** |
| **hsa-miR-376a** | **hsa-miR-181c** |
| **hsa-miR-342** | **hsa-miR-409-3p** |
| **hsa-miR-323** | **hsa-miR-504** |
