# Supplementary material for: Surprisal Analysis of Glioblastoma Multiform (GBM) MicroRNA Dynamics Unveils Tumor Specific Phenotype
Source: PLoS One. 2014 Sep 29;9(9):e108171. doi: 10.1371/journal.pone.0108171 (PMC4180445; doi:10.1371/journal.pone.0108171)
Supplement: Table S3 — miRNAs greatest positive free energy contribution to the GBM-specific phenotypic state. (DOCX) [file pone.0108171.s004.docx]

**Table S3:** The miRNAs with the greatest positive free energy contribution to the *GBM-specific phenotypic state.* MiRNAs are listed in descending order down the column and continues from left column to right column. (G1 up-regulated)

| **hsa-miR-21** | **hsa-miR-148a** |
| --- | --- |
| **hsa-miR-210** | **hsa-miR-142-5p** |
| **hsa-miR-27a** | **hsa-miR-663** |
| **hsa-miR-23a** | **hsa-miR-92** |
| **hsa-miR-155** | **hsa-miR-182** |
| **hsa-miR-142-3p** | **hsa-miR-339** |
| **hsa-miR-199a*** | **hsa-miR-513** |
| **hsa-miR-370** | **hsa-miR-17-5p** |
| **hsa-miR-15b** | **hsa-miR-195** |
| **hsa-miR-25** | **hsa-miR-452** |
| **hsa-miR-106b** | **hsa-miR-30a-5p** |
| **hsa-miR-214** | **hsa-miR-188** |
| **hsa-miR-223** | **hsa-miR-19a** |
| **hsa-miR-630** | **hsa-miR-494** |
| **hsa-miR-801** | **hsa-miR-19b** |
| **hsa-miR-34a** | **ebv-miR-BART19** |
| **hsa-miR-15a** | **hsa-miR-424** |
| **hsa-miR-130b** | **hsa-miR-296** |
| **hsa-miR-20a** | **hsa-miR-10b** |
| **hsa-miR-146b** | **kshv-miR-K12-3** |
| **hsa-miR-106a** | **hsa-miR-638** |
| **hsa-miR-199a** | **hsa-let-7i** |
| **hsa-miR-93** | **hsa-miR-493-3p** |
| **hsa-miR-16** | **hsa-miR-92b** |
| **hsa-miR-193a** | **hsa-miR-320** |
