# Supplementary material for: Surprisal Analysis of Glioblastoma Multiform (GBM) MicroRNA Dynamics Unveils Tumor Specific Phenotype
Source: PLoS One. 2014 Sep 29;9(9):e108171. doi: 10.1371/journal.pone.0108171 (PMC4180445; doi:10.1371/journal.pone.0108171)
Supplement: Table S4 — Overlap of signatures between UCLA GBM Cohort and TCGA GBM Cohort. (DOCX) [file pone.0108171.s005.docx]

**Table S4:** Surprisal analysis reveal consistent signatures between TCGA and UCLA patient cohorts. MiRNAs are listed in ascending order down the column and continues from left column to right column. Overlap of miRNAs between the two cohorts was color-matched to highlight the similarity.

| **TGCA Patient Cohort** | **UCLA Patient Cohort** |
| --- | --- |
| **hsa-miR-21** | **hsa-miR-923** |
| **hsa-let-7b** | **hsa-let-7a** |
| **hsa-let-7a** | **hsa-miR-125b** |
| **hsa-miR-9*** | **hsa-let-7b** |
| **hsa-miR-125b** | **hsa-let-7f** |
| **hsa-let-7c** | **hsa-let-7c** |
| **hsa-miR-29a** | **hsa-miR-21** |
| **hsa-miR-26a** | **hsa-miR-29a** |
| **hsa-let-7f** | **hsa-miR-26a** |
| **hsa-miR-9** | **hsa-miR-9** |
| **hsa-let-7e** | **hsa-miR-451** |
| **hsa-miR-24** | **hsa-miR-9*** |
| **hsa-let-7d** | **hsa-miR-16** |
| **hsa-miR-22** | **hsa-miR-494** |
| **hsa-miR-126** | **hsa-let-7g** |
| **hsa-miR-16** | **hsa-let-7i** |
| **hsa-miR-195** | **hsa-miR-99a** |
| **hsa-let-7g** | **hsa-miR-100** |
